# Unexpected esophageal diseases appeared in thyroid resections

**DOI:** 10.1186/s12957-015-0542-5

**Published:** 2015-03-30

**Authors:** Liu Ye-huan, Lyu Shi-xu, Zhou Yi-li, Wang Ou-chen, Zhang Xiao-hua

**Affiliations:** Department of Oncology, The First Affiliated Hospital of Wenzhou Medical University, South of Bai-xiang Street, Ou-hai District, 325000 Wenzhou, Zhejiang People’s Republic of China

**Keywords:** Cervical esophageal cancer, Zenker’s diverticulum, Thyroid misdiagnosis

## Abstract

**Objective:**

In order to avoid the misdiagnosis of thyroid diseases, we need to discuss the clinical features and diagnostic methods of cervical esophageal cancer and Zenker’s diverticulum.

**Methods:**

The clinical and laboratory data of seven cases were reviewed retrospectively, and in all cases, esophageal-related diseases were misdiagnosed as thyroid diseases preoperatively. Among them, two cases were cervical esophageal cancer metastasized to thyroids but initially, they were misdiagnosed as thyroid cancer. The other five cases were Zenker’s diverticulum, but were originally diagnosed as nodular goiter, and two out of the five cases were found with calcification. They were all detected by ultrasound examination without any clinical feature of esophageal diseases. Previous literatures only reported five cases of thyroid metastasis and three cases of Zenker’s diverticulum.

**Results:**

In both cases where cervical esophageal cancer metastasized to thyroid, anterior cervical neoplasm biopsy and surgical removal were performed followed by postoperative radiotherapy and chemotherapy. Both patients died from esophageal cancers in 7 and 15 months postoperatively. All five cases of Zenker’s diverticulum received excision and repair without any postoperative complication or recurrence in the following 2 to 7 years.

**Conclusions:**

Cervical esophageal cancer and Zenker’s diverticulum may be misdiagnosed as thyroid disease. Careful and comprehensive diagnostic tests would be required to avoid misdiagnosis.

## Background

Thyroid diseases are commonly encountered endocrine disease in clinics. However, symptoms from other neck neoplasms such as esophageal diseases can mimic thyroid diseases sometimes and lead to misdiagnosis. With rising public awareness of primary prevention, neck ultrasound has been widely used as a routine project for thyroid abnormality [1]. About 3,000 cases of thyroid resection were performed in oncological surgery at our hospital every year. Among them, seven cases, collected from May 2006 to October 2014, were analyzed here, in which all esophageal-related diseases were misdiagnosed as thyroid diseases preoperatively.

## Case presentation

### Clinical data

#### Physical examination

The neck neoplasms in all cases moved with swallowing, and they were medium-hard texture. No patients reported sensation of dysphagia or reflux, and no neck lymph node enlargement was appreciated. One of two patients with metastatic cervical esophageal cancer to the thyroid had obvious hoarseness, so did one patient with Zenker’s diverticulum. The rest of Zenker’s diverticulum all had anterior cervical pressure sensation.

#### Laboratory examinations

**Figure 1:**
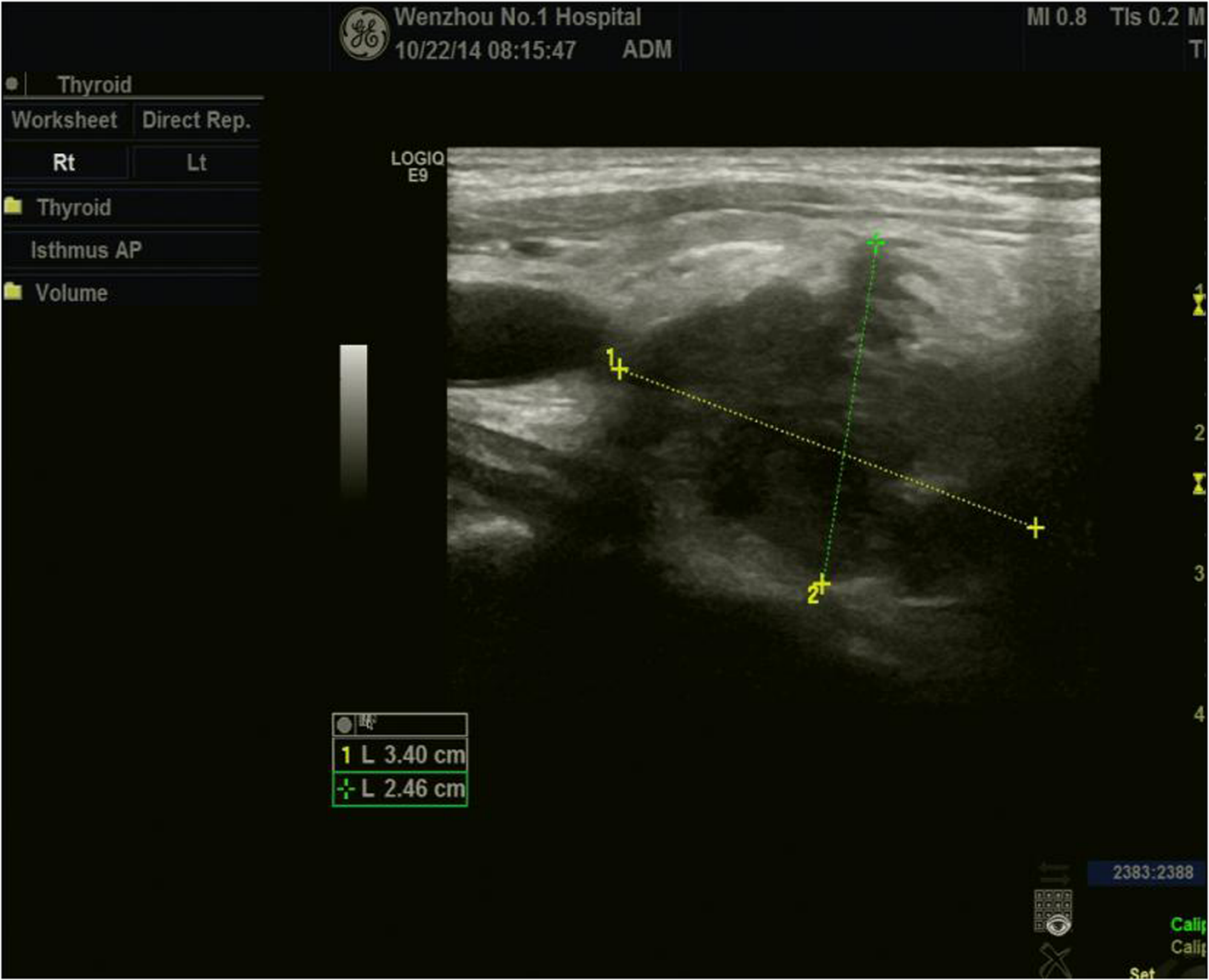
**Ultrasound reexamination in a week before death showed a solitary, irregular, hypoechoic mass with some hyperechoic foci.** Its border was unclear and the max diameter was 3.4 cm.

**Figure 2:**
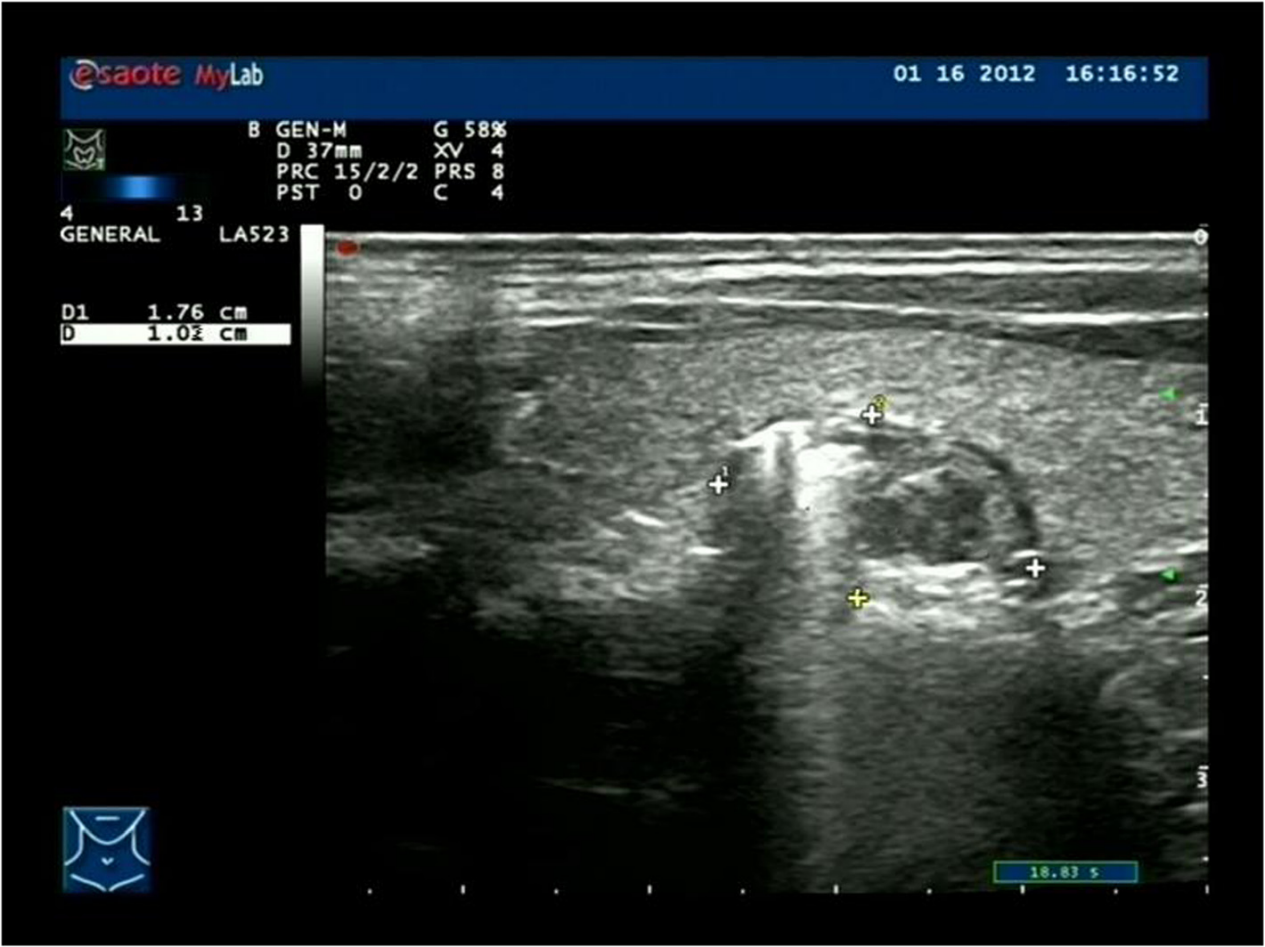
**Ultrasound examination showed a solitary, irregular, hypoechoic of mass with post acoustic shadow.** Its border was clear and smooth with a max diameter 1.8 cm.

**Figure 3:**
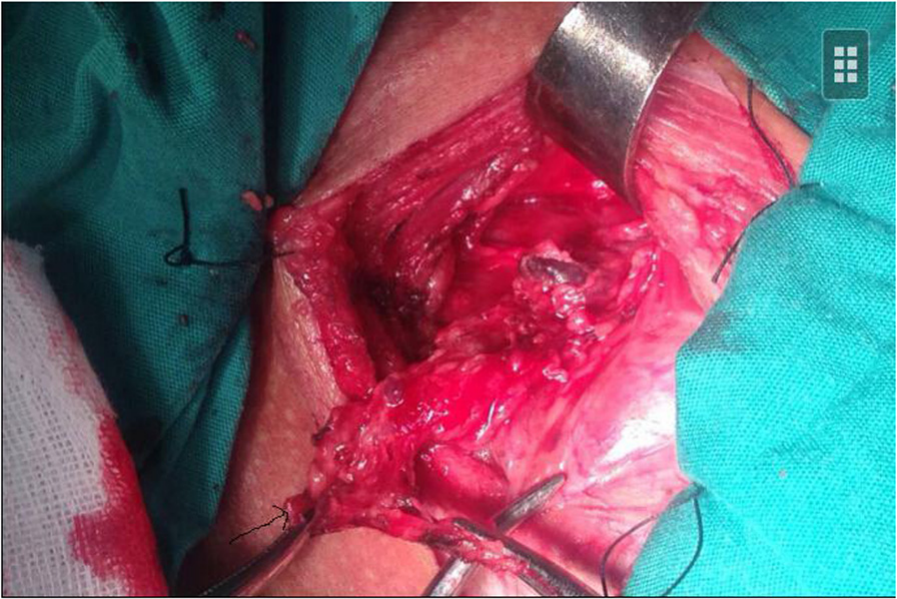
**Neoplasm (arrowheads) attached to thyroid and invaded the right recurrent laryngeal nerve.**

**Figure 4:**
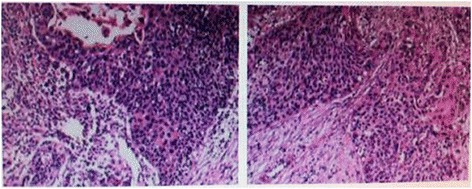
**Pathological examination showed high differentiated squamous carcinoma.**

**Figure 5:**
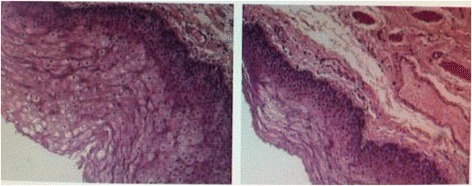
**Pathological examination showed squamous epithelium with some small glands in lamina propria.**

Thyroid hormone series and antibody were measured in seven cases. TSH, TT3, TT4, FT3, and FT4 were all in normal range. Only one case of esophageal diverticulum had mildly elevated TgAb concentration (12.1 IU/ml with normal value approximately 0 to 4 IU/ml) as shown in Table 1. Thyroid ultrasound (Figures 1 and 2) was performed in all seven cases in which three cases accepted FNA, shown in Table 2. Total thyroidectomy with possible lymphadenectomy was originally planned for all seven cases. Display of surgery (Figure 3) amazed us. However, as intraoperative frozen biopsies (Figures 4 and 5) proved accurate by postoperative histopathological examinations in the following days altered the initial diagnosis, surgical plan was changed depending on the individual pathological result in each case after emergent thoracic surgery consultation. Details are in Table 3.

**Table 1:** **Physical and laboratory examinations**

**Cases**	**Age (years)/sex**	**Esophageal disease**	**Symptoms**	**Size (cm)**	**Location**	**Thyroid hormone and related antibody**
1	54/M	Cervical esophageal cancer	No	5 × 2.3	Behind the right lobe	Normal
2	50/M	Cervical esophageal cancer	Hoarseness	3.4 × 2.5	Behind the right lobe	Normal
3	47/F	Zenker diverticulum	Pressure sensation	2.2 × 4	Behind the left lobe	Normal
4	39/F	Zenker diverticulum	Pressure sensation	3.2 × 3.6	Behind the left lobe	Normal
5	54/M	Zenker diverticulum	Pressure sensation	1.8 × 1	Behind the left lobe	Normal
6	35/F	Zenker diverticulum	Pressure sensation	4 × 3.5	Behind the left lobe	Normal
7	37/F	Zenker diverticulum	Hoarseness	4 × 4	Behind the left lobe	TgAb↑

**Table 2:** **Ultrasound and FNA**

**Cases**	**Ultrasound description**	**Misdiagnosis**	**FNA**
1	Solitary, hypoechoic mass with hyperechoic foci	Thyroid nodule TI-RADS IVc	+^a^
2	Solitary, hypoechoic mass with hyperechoic foci (Figure 1)	Thyroid nodule TI-RADS IVb	+^b^
3	Solitary, irregular hyperechoic area	Nodular goiter	No
4	Solitary, complex hypoechoic mass	Nodular goiter	No
5	Solitary, hypoechoic of mass with post acoustic shadow (Figure 2)	Nodular goiter with calcification	No
6	Solitary, patchy hyperechoic mass	Nodular goiter	No
7	Solitary, nodular calcification	Nodular goiter with calcification	−^c^

**Table 3:** **Intraoperative frozen biopsy and type of surgery**

**Cases**	**Display of surgery**	**Intraoperative frozen biopsies**	**Type of surgery**
1	Neoplasm adhered to thyroid to form a firm mass which also enclosed the internal jugular vein.	Poorly differentiated squamous carcinoma	Anterior cervical neoplasm biopsy^a^
2	Neoplasm attached to thyroid and invaded the right recurrent laryngeal nerve. (Figure 3)	Highly differentiated squamous carcinoma (Figure 4)	Anterior cervical neoplasm biopsy^a^
3	Neoplasm was cystic with integrated envelope and its central cavity communicated with esophagus.	Zenker’s diverticulum^b^	Excision and repair
4	Neoplasm protruded from esophagus with food remains in it.	Zenker’s diverticulum^c^	Excision and repair
5	Neoplasm can be touched from esophageal outer membrane and the texture was soft.	Zenker’s diverticulum^d^ (Figure 5)	Excision and repair
6	Neoplasm was pouch-like and communicated with esophageal pyriform sinus.	Zenker’s diverticulum^e^	Excision and repair
7	Neoplasm compressed the left laryngeal recurrent nerve.	Zenker’s diverticulum^f^	Excision and repair

^a^Based on the consultations of thoracic surgeons and histopathological examinations, we performed the surgery and took a little of tissue sample for biopsy in order to avoid the esophageal fistula and unnecessary damage. Therefore, the majority of neoplasm is remaining and the size is similar with previous. ^b^Squamous epithelium has hyperplasia with erosion and chronic inflammatory cells invade the lamina propria. Hemangiectasis is obvious. ^c^Squamous epithelial mucosa is chronically inflammatory and the base layer cracks have no cell and other ingredients in it. ^d^The lining of cystic tissue wall is squamous epithelium with some small glands in lamina propria. ^e^Mucosal surface concave into cavity with squamous epithelium as lining. ^f^Squamous epithelial has significant hyperplasia.

All the ultrasound descriptions were based on the preoperative records of the ultrasound examinations. ^a^Many thyroid follicular epithelial cells, several abnormal cells with intranuclear inclusions, sporadic polynuclear giant cells. ^b^Many thyroid follicular epithelial cells with bits of allotypic cells between focal areas of fibrous tissue. ^c^No obvious allotypic cells and sporadic inflammatory cells in pectin background.

### Follow-up data

Both cases of metastatic cervical esophageal cancer to thyroid received nasal feeding for 1 week postoperatively, and no esophageal fistula occurred. Later, they accepted radiotherapy and chemotherapy according to the 2011 National Comprehensive Cancer Network (NCCN) Esophageal Cancer Guidelines. They died from esophageal cancer in 7 and 15 months postoperatively. All five cases of Zenker’s diverticulum had no postoperative complications or recurrence in the following 2 to 7 years and survived to the present.

## Discussion

The incidence of metastatic spread of gastrointestinal malignancies to the thyroid gland is relatively low, and most of them are from the colo-rectum [2]. Thyroid metastasis originating from the esophagus is poorly documented. We conducted a review of current English literature related to such condition, and there have been a total of five cases reported previously [3-7]. Here, we presented two additional cases of thyroid metastasis from cervical esophageal cancer.

Table 4 summarized the clinical circumstances and ultrasound results from the five cases previously published plus our two cases of thyroid metastasis from cervical esophageal cancer. Among the seven patients, two patients were women and five were men. Their mean age was 55 years, with a range from 32 to 74 years. The majority of patients underwent thyroidectomy. The postoperative histopathological examination all showed squamous cell carcinoma. Most of the patients with thyroid metastasis had a poor prognosis and died shortly after diagnosis. Details are in Table 4.

**Table 4:** **Clinical circumstances and ultrasound examinations**

**Source location**	**Age (years)/sex**	**Type of surgery (months)** ^**a**^	**Outcomes**	**Size (cm)**	**Ultrasound**	**Description**
Case 1	54/M	Anterior cervical	7	5 × 2.3	Solitary, hypoechoic mass	R
		Neoplasm biopsy			With hyperechoic foci	R
Case 2	50/M	Anterior cervical	15	3.4 × 2.5	Solitary, hypoechoic mass	
		Neoplasm biopsy			With hyperechoic foci	
En-dong [3]	61/M	Palliative bilateral NT + tracheostomy	11	6.1 × 3.9	Solitary mass, heterogeneous, hypoechoic	L
Shuangshoti S et al. 1982 [4]	58/M	TT + ipsilateral CL	5	1.5 × 1.5	Solitary mass, NA	R
Yamada T et al. 1999 [5]	74/F	ST + Bilateral CL	NA	NA	Widespread masses, calcified	Not specified
Basu S et al. 2005 [6]	55/F	NA	NA	6 × 4	Solitary mass, irregular, hypoechoic	R
Cumbo-Nacheli G et al. 2007 [7]	32/M	NA	NA	2.5 × 2.8	Solitary mass, NA	R

^a^Follow-up since diagnosis of intra-thyroid metastases. NA, no data available; NT, near-total thyroidectomy; ST, subtotal thyroidectomy; TT, total thyroidectomy; CL, cervical lymphadenectomy.

The incidence of Zenker’s diverticulum mimic thyroid nodules is poorly documented. We conducted a review of the English and Chinese literature related to such condition and there were three published cases [8-10]. This article presents five additional case of Zenker’s diverticulum mimic thyroid nodules.

Table 5 summarizes the clinical circumstances and ultrasound examination results for the three cases previously published plus our report of Zenker’s diverticulum mimic thyroid nodules. Of the eight patients with Zenker’s diverticulum, seven patients were women and one was a man. Their mean age was 49 years old, with a range of 35 to 73 years. Based on the above chief complaint, apart from some mild pressure sensation and foreign body sensation, all patients were asymptomatic and were not experiencing dysphagia, difficulty in swallowing, or reflux. Six patients underwent excision and repair and were recovered. Our patients are alive while there is no available follow-up data for the others. All the Zenker’s diverticulum were located in the posterior aspect of the left thyroid lobe. Details are in Table 5.

**Table 5:** **Clinical circumstances and ultrasound examinations**

**Source**	**Age (years)/sex**	**Chief complaint**	**Type of surgery**	**Size (cm)**	**ultrasound description**	**Location**
Case 3	47/F	Pressure sensation	Excision and repair	2.2 × 4	Solitary, irregular hyper echoic area	L
Case 4	39/F	Pressure sensation	Excision and repair	3.2 × 3.6	Solitary, substantial low echo	L
Case 5	54/M	Pressure sensation	Excision and repair	1.8 × 1	Solitary, low echo of mass with post acoustic shadow	L
Case 6	35/F	Pressure sensation	Excision and repair	4 × 3.5	Solitary, hyper echoic foci	L
Case 7	37/F	Hoarseness	Excision and repair	4 × 4	Solitary, grit calcification	L
Bin [8]	50/F	Mild pharyngeal foreign body sensation	No	1.2 × 0.6	Solitary, hypo echoic, calcified	L
Yong Fang et al. 2011 [9]	73/F	Finding left neck mass	Excision and repair	3 × 1.8	Solitary, cystic and solid mass	L
Beth-Ann [10]	54/F	Finding left neck mass	NA	2 × 1.2	Solitary, heterogeneous hypo echoic	L

NA, no data available.

A Zenker’s diverticulum is a herniation of the mucosa and submucosa at Killian’s triangle, a natural area of weakness at the junction of the thyropharyngeus and cricopharyngeus muscles in the posterior hypopharynx. It is believed that these diverticula are pulsion diverticula occurring as a result of spasm of the cricopharyngeus muscle, in coordination of the pharyngeal muscles or congenital muscle weakness. Due to the weak area is more obvious in the left side, Zenker’s diverticula project to the left [11-16].

On ultrasonography, we can examine the sonographic similarities and differences between a Zenker’s diverticulum versus a true thyroid abnormality as the following points. First, heterogeneous internal echo with strong echogenic foci caused by air bubbles or other particles could be regarded as micro calcifications of thyroid cancer, but there is stronger echogenicity and irregularity of the post acoustic shadow. Second is a hypo echoic rim with or without a multilayered pattern. This finding suggests that the digestive tract is the origin of the lesion (mucosa, submucosa, and muscular layers).Third is an irregular boundary of the posterior wall of the lesion at the posterior portion of the thyroid gland. Fourth are the chronological changes in the internal echo which are associated with changes in the contents of the diverticulum, such as air, water, or debris. These changes result during compression with a probe or during the swallowing of air or water [17-22].

According to our cases, some perspectives about misdiagnosis on esophageal diseases can be analyzed. First, they were all lacking of typical clinical symptoms such as dysphagia and reflux, so it is difficult to be detected in early stage at the most of the time [23,24]. Second, neoplasms moved by swallowing were all located closely in the posterior aspect of the thyroid gland and present as thyroid abnormality on ultrasonography. Third, because of the similar location, fine-needle aspiration inevitably brought out bits of thyroid cells so that it is too difficult to distinguish between primary and metastatic thyroid malignancies when highly anaplastic cells are observed microscopically [25]. Even though in our cases, we get the misleading FNA resulted by inadequate specimen, there are still studies to prove the false-negative rate of FNA is less than 1% and false-positive rate is only 1% to 3% in thyroid diagnosis [26-28]. In addition, according to the previous reports, FNA can improve the diagnosis of thyroid carcinoma and total diagnostic accuracy is 87.5%, diagnostic accuracy of benign lesions is 93.8%, and the diagnostic accuracy of malignancies is 97.3% in cervical masses [29-32].Besides, FNA is also fast, safe, and convenient which has been considered as a gold standard second to histopathological examinations.

Then, what should we do to avoid the misdiagnosis between esophageal and thyroid lesions? Take the medical histories and physical examinations carefully and especially pay attention to the special clinical symptoms of esophageal diseases such as dysphasia or reflux [12,16].According to the 2010 National Comprehensive Cancer Network (NCCN) Thyroid Carcinoma Guidelines, measuring TSH and accepting the ultrasonography were considered as routine projects in thyroid diseases. To the suspected thyroid malignancies after ultrasonography, FNA is often recommended. In order to improve the diagnostic accuracy, we can puncture and smear more to get satisfactory specimens. When it illustrates that sonographic left-sided thyroid nodules that exhibit squamous cells, bacteria, or foreign material on FNA biopsy, we should raise the suspicion of an occult Zenker’s diverticulum. What is more, X-ray barium meal examination, endoscopy, ECT, CT, MRI, and CNB could be applied to help to make the correct diagnosis if necessary [33-41].

## Conclusions

Cervical esophageal cancer and Zenker’s diverticulum may be misdiagnosed as thyroid diseases. Careful and comprehensive diagnostic tests would be required to avoid misdiagnosis.

## Consent

Written informed consent was obtained from the patients for publication of this case report and any accompanying images. A copy of the written consent is available for review by the Editor-in-Chief of this journal.

## Abbreviations

TSH: thyroid stimulating hormone
FNA: fine-needle aspiration
ECT: emission computed tomography
CT: computed tomography
MRI: magnetic resonance imaging
CNB: core needle biopsy
